# *N*-acetylcysteine mitigates acute opioid withdrawal behaviors and CNS oxidative stress in neonatal rats

**DOI:** 10.1038/s41390-019-0728-6

**Published:** 2020-01-14

**Authors:** Price Ward, Hunter G. Moss, Truman R. Brown, Peter Kalivas, Dorothea D. Jenkins

**Affiliations:** 1 Department of Pediatrics, Medical University of South Carolina, Charleston, SC USA; 2 Department of Neuroscience, Medical University of South Carolina, Charleston, SC USA; 3 Department of Radiology, Medical University of South Carolina, Charleston, SC USA

## Abstract

**Background:**

Neonatal abstinence syndrome (NAS) is a significant problem. Opioid withdrawal induces oxidative stress and disrupts glutamate and glutathione homeostasis. We hypothesized that *N*-acetylcysteine (NAC) administered during acute opioid withdrawal in neonatal rats would decrease withdrawal behaviors and normalize CNS glutathione and glutamate.

**Methods:**

Osmotic minipumps with methadone (opioid dependent, OD) and saline (Sham) were implanted into Sprague Dawley dams 7 days prior to delivery. Pups were randomized to receive either naloxone plus saline or NAC (50–100 mg/kg), administered on postnatal day (PND) 7. We performed MR spectroscopy on PND6–7 before, 30 min, and 120 min after withdrawal. On PND7, we assessed withdrawal behaviors for 90 min after naloxone administration and summed scores during peak withdrawal period.

**Results:**

Mean summed behavioral scores were significantly different between groups (*χ*^2^ (2) = 10.49, *p* = 0.005) but not different between NAC/NAL/OD and Sham (*p* = 0.14): SAL/NAL/OD = 17.2 ± 4.2 (*n* = 10); NAC/NAL/OD = 11.3 ± 5.6 (*n* = 9); Sham = 6.5 ± 0.6 (*n* = 4). SAL/NAL/OD pups had decreased glutathione at 120 min (*p* = 0.01), while NAC/NAL/OD pups maintained pre-withdrawal glutathione (*p* = 0.26).

**Conclusion:**

In antenatal OD, NAC maintains CNS glutathione and mitigates acute opioid withdrawal in neonatal rats. This is the first study to demonstrate acute opioid withdrawal neurochemical changes in vivo in neonatal OD. NAC is a potential novel treatment for NAS.

## Introduction

Neonatal abstinence syndrome (NAS) occurs in neonates after birth who have been exposed to opioids in utero. From 2000 to 2012, there was a fivefold increase in the United States in the number of babies who developed NAS. Moreover, from just 2009 to 2012 the incidence of NAS has increased from 3.4 to 5.8 per 1000 hospital births with an estimated 21,000 infants with NAS in 2012,^1,2^ suggesting the problem continues to worsen. In addition, neonates being treated for NAS stayed on average 23 days in the hospital (compared to 2.1 days for other newborns of comparable gestational age), often in a neonatal intensive care unit.^1^ Hospitals incurred $1.5 billion in associated costs, with 81% of these costs borne by state Medicaid programs.^1^ Currently, the mainstay of treatment is using either morphine or methadone, occasionally in combination with clonidine or phenobarbital.^3–5^ Despite the early implementation of both pharmacologic and non-pharmacologic treatments, NAS continues to be a prolonged and difficult management problem for healthcare teams.

NAS is characterized by vital sign changes, tremors, excessive crying, hyperirritability, gastrointestinal disturbances, and feeding intolerance.^3–5^ These symptoms are likely due to the effects of increased production of neurotransmitters dopamine, norepinephrine, acetylcholine, and serotonin,^4,6^ which act on the locus coeruleus, ventral tegmental area, and nucleus accumbens.^4,6,7^ Opioid replacement therapy not only mitigates these changes in neurotransmitters but is also associated with apoptosis, white and grey matter injury, altered redox homeostasis, and changes in oligodendroglial maturation and myelination, which may be particularly damaging in the developing brain.^8–13^ To counteract the excess neurotransmitter release and yet avoid opioid replacement, investigators have taken several approaches that may apply to neonatal abstinence. In an adult opioid-dependent animal model, other investigators have determined that withdrawal precipitates oxidative stress, and measures of oxidative stress directly relate to withdrawal behaviors, which are responsive to antioxidants.^14–16^ In addition to oxidative stress, there is a disruption of glutamate homeostasis during opioid withdrawal resulting in an increase in synaptic glutamate (excitatory neurotransmitter) that leads to increased neuronal firing.^17–21^ This increase in synaptic glutamate occurs because of decreased tone and function of the extrasynaptic metabotropic glutamate autoreceptors, mGluR2/3, which normally inhibits synaptic glutamate release (this decreased inhibition leads to increased synaptic glutamate)^17,20^ and decreased function of glial glutamate transporter-1, which causes reduced glutamate elimination from extracellular space resulting in spillover of synaptic glutamate.^17,20^

Ongoing research in both humans and animals are investigating restoring the glutamate and redox imbalance with *N*-acetylcysteine (NAC).^14–16,20^ NAC is a cysteine prodrug and glutathione precursor, which enters glial cells in exchange for glutamate and is converted to intracellular glutathione, a potent antioxidant and neuroprotective substrate. NAC inhibits both the decrease in glutathione and withdrawal behaviors in an adult animal model of heroin dependence.^14^ In addition, NAC restores the function of mGluR2/3 receptors, cysteine–glutamate exchanger, and glutamate transporter-1, thus re-establishing glutamate homeostasis.^20,22^ Research in adult rats suffering from morphine withdrawal has shown that mGluR2/3 agonists attenuate symptoms of opioid withdrawal.^21,23,24^ Furthermore, extensive research in adults with substance use disorders, specifically nicotine, cocaine, and the opioid heroin, demonstrate that NAC reduces cravings and cue reactivity.^20,25^

Currently, no known studies have investigated the effects of NAC on opioid withdrawal in neonates. The aims of this study were to establish a rat model of antenatal opioid dependence and neonatal acute opioid withdrawal (Experiment 1) and to evaluate the effects of NAC on acute opioid withdrawal in the neonatal rat on outcomes of withdrawal behavioral scores and central nervous system (CNS) metabolism (Experiment 2). To characterize the effect of acute opioid withdrawal and NAC administration on CNS metabolism, we utilized in vivo magnetic resonance spectroscopy (MRS) to determine the concentrations of metabolites in the neonatal rat brain before and after acute opiate withdrawal, including glutathione (GSH), glutamate-glutamine (GLX), *N*-acetylaspartate (NAA), and creatine (Cr). We hypothesized that NAC administered during acute opioid withdrawal would decrease withdrawal behaviors, normalize [GLX], and increase [GSH] and [NAA] concentrations in the neonatal rat brain, thus providing neuroprotection from the oxidative stress of acute withdrawal.

## Methods

### Animals

Timed-pregnant Sprague Dawley rats (Envigo Indianapolis, IN, USA) were delivered on gestational day (GD) 13. Animals were housed in our animal facility with temperature maintained at 22–24 °C, a 12-h light–dark photocycle with light onset at 0700 hours and ad libitum access to food and water. This study was conducted in accordance with the National Institute of Health *Guide for the Care and Use of Laboratory Animals*. The protocol was approved by the Institutional Animal Care and Use Committee at The Medical University of South Carolina (MUSC).

### Opioid exposure

Prenatal opioid exposure was achieved using methadone hydrochloride. Methadone was chosen over morphine due to its longer half-life, which eliminates the needs to replace morphine pellets or re-dose morphine postnatally. Although buprenorphine has been used in animal studies, they are less extensive and have limited pharmacokinetics.^26^ On GD 15, we prepared a 28-day Model 2ML4 osmotic minipump (Alzet®, Palo Alto, CA, USA) to deliver methadone hydrochloride (9 mg/kg/day) for a steady-state blood level^13,27,28^ or saline (equivalent volume). It was primed overnight in sterile 0.9% saline at 37 °C, according to the manufacturer’s recommendations. Methadone was generously supplied by the National Institute on Drug Abuse (NIDA, Rockville, MD). After a 72-h acclimation period, the minipump was implanted subcutaneously (s.c.) in GD 16 dams, under isoflurane anesthesia (4–5% for induction; 1–3% for maintenance) and carprofen (5 mg/kg, s.c.) for analgesia. After recovery, the dams were placed back into the colony room where they were assessed daily until delivery on GD 21–23. Within 24 h of parturition, litters were culled to a maximum of 10 offspring and pups were weighed and randomly assigned a study ID and treatment group. Methadone exposure continued via ad libitum breast milk^13,27,28^ until postnatal days (PNDs) 6–7 when methadone- and saline-exposed pups underwent acute or sham withdrawal, behavioral testing, and MRS.^26,29–31^

### Acute precipitated withdrawal

In Experiment 1, to establish the model and validate the behavioral scoring, pups were randomized within litter to receive either naloxone hydrochloride (1 mg/kg, intraperitoneal (i.p.)), a dose that has been shown to precipitate acute withdrawal in rats,^26,29–31^ or an equal volume of saline. Treatment groups in Experiment 1 were Sham (pups with neither methadone exposure nor naloxone), SAL OD (pups with methadone exposure, opioid dependent, and saline but no naloxone), and NAL OD (methadone exposure, opioid dependent, and naloxone).

In Experiment 2, to determine the effects of NAC on behavioral scores and CNS metabolites after acute withdrawal, all methadone-exposed pups received naloxone HCl (1 mg/kg, i.p.) and either NAC (50 mg/kg, *n* = 2, or 100 mg/kg, *n* = 7) or an equal volume of saline i.p. Pups were randomized within litter based on maternal exposure to either methadone or saline. Treatment groups in Experiment 2 were SAL/NAL OD (methadone exposed, opioid dependent, naloxone and saline) and NAC/NAL OD (methadone exposed, opioid dependent, naloxone, and NAC). Sham (neither methadone exposed nor naloxone) were used as behavioral controls.

### Behavioral testing

Behavioral testing was performed on PND 7. Two pups were injected with saline or naloxone and then moved to separate plastic observation chambers (11 cm × 30 cm × 11 cm) heated with small animal heating pads to maintain body temperature while away from the litter and dam. Chamber temperatures were continuously monitored and regulated during the observation period to keep body temperatures at 36 °C (Supplementary Table S1) at the start and end of the behavior testing. Behaviors were continuously recorded from 0 up to 90 min after naloxone/saline administration. Withdrawal behaviors, adapted from Barr et al.,^28,32,33^ included paw movement, rolling, stretching, body curls, and mild and severe tremors (Table 1), were observed for 30-s intervals every 5 min, and recorded on a checklist. All of the behaviors (except for mild and severe tremors) were given one point for every one occurrence (i.e., 5 body curls in a 30-s period = score of 5) over the 30-s interval. Mild and severe tremors were tallied for every one occurrence and then summed. A scaled scoring system was then used where 0–2 tallies = score of 1; 3–5 tallies = score of 2; 6–8 tallies = score of 3; and ≥9 tallies = score of 4. For each time period, scores for each behavior were summed to get a total score for that given time period and then summed for the duration of the observation period to get the total summed withdrawal behavior score (withdrawal score). In Experiment 1, behaviors were scored from 0 to 60 min. At 60 min, however, behavior scores were still increasing, indicating peak withdrawal was occurring beyond the 60 min of observation. Therefore, in Experiment 2, behaviors were observed for 90 min, and we determined the peak withdrawal period from 35 to 75 min. Withdrawal scores were summed in Experiment 2 from 35 to 75 min.^30,33^ Two pups were tested at a time (videotaped individually), and videotapes were scored by a blinded observer.

**Table 1:** Behaviors of opioid withdrawal.

Behavior	Description	Scoring
Paw movement	Continuous movement of the paws without walking	1 point for every occurrence
Rolling	Turning the body over (to a side or complete roll)	1 point for every occurrence
Stretch	Extension or dorsal flexion of the trunk; elongation of the extremities	1 point for every occurrence. If unsure stretch vs curl, point given to stretch. Do not double count
Body curls	Ventral or lateral flexion of the trunk	1 point for every occurrence. See above
Mild tremors	Lateral head shakes	1 tally for every tremor. Scaled scoring: 0–2 tallies = score of 1; 3–5 tallies = score of 2; 6–8 tallies = score of 3; 9+ tallies = score of 4
Severe tremors	Full body shakes	1 tally for every tremor. Scaled scoring: 0–2 tallies = score of 1; 3–5 tallies = score of 2; 6–8 tallies = score of 3; 9+ tallies = score of 4

Adapted from Barr et al.^29,33,34^

### Magnetic resonance spectroscopy

**Fig. 1:**
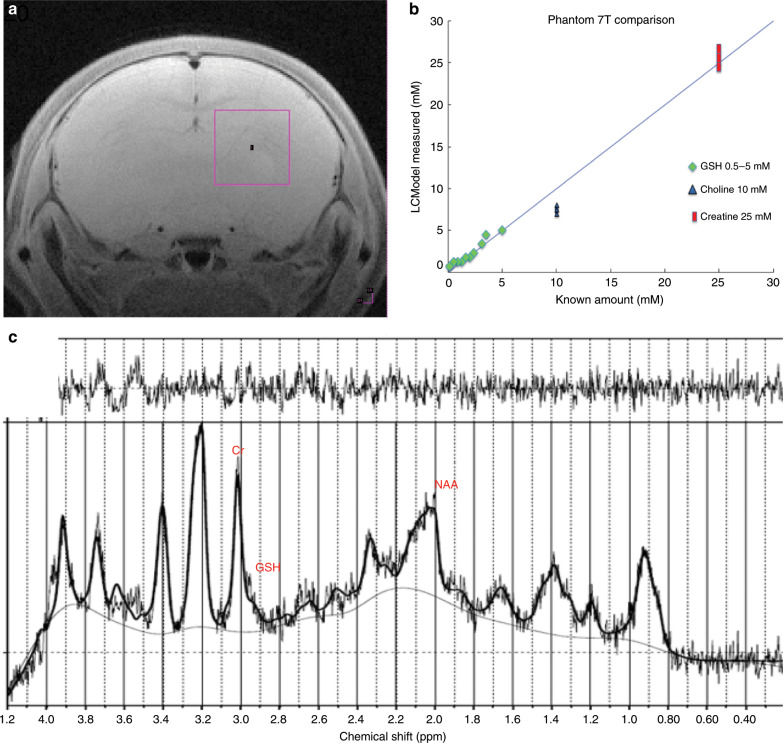
MRS data processing. **a** T2 image with anatomical placement of the voxel in the right hemisphere; **b** phantom solution standard curve; **c** representative spectra output from LCModel.

In Experiment 2 to establish the effects of opioid withdrawal and NAC on CNS metabolism, we performed MRS scans on PND 6 and 7 prior to precipitated withdrawal (Pre-scans) and on PND 7 following the acute precipitated withdrawal, at 30 and 120 min (Post-scans). Owing to time constraints induced by scheduling of MRS before and after the behavioral observation period, not all pups in a litter could be scanned at both 30 and 120 min. Pups were anesthetized with isoflurane 1–2% mixed with warm compressed air/oxygen for the MRS scans, then placed on a MR compatible, heated animal bed (temperature maintained at 38.0 ± 0.2 °C) connected to an animal monitoring unit (SA Instruments, Inc., Stony Brook, NY), which recorded respirations, pulse oximetry, and core temperature. We used a 7 Tesla (T) BioSpec 70/30 horizontal magnetic resonance imaging (MRI) scanner (Bruker^©^ BioSpin, Ettlingen, Germany). The system is equipped with a 12-cm gradient and shim coil set, capable of generating maximum gradient amplitude of 400 mT/m and 4-channel receiver for multi-coil operation. High-resolution T2-weighted anatomy scans were performed for anatomical positioning of the voxel (Fig. 1a). After reconstruction and voxel placement (3 × 3 × 3 mm^3^), an MRS water reference was acquired for each animal, followed by STEAM sequence (TR 1500 ms, TE = 3 ms, TI = 10 ms, spatial-water-fat shift = 0.467 mm, spectroscopy number of points = 2048, spectral width = 4006.41 Hz, water suppression scheme = VAPOR, bandwidth 200 Hz, outer volume suppression = ON, OVS slice thickness = 10 mm, gap to voxel = 0 mm, number of averages = 512, scanning time = 12.8 min) in the right hemisphere. At the conclusions of all experiments, animals were euthanized according to the euthanasia protocol at MUSC.

### MRS data processing

Phantom solutions (0.5–5 mM GSH, 5 mM DTT, 10 mM Choline, 25 mM Creatine, phosphate-buffered saline, pH 7.1) were used to validate [GSH], [Cr], and [Cho] quantification by MRS, using the water peak as a standard.^34^ Quantification of GSH and Cr in phantoms by LCModel processing with our specialized basis set showed excellent correlation with known concentrations of these metabolites.^34^ The phantom solution standard curves for [GSH], [Cho], and [Cr] are shown in Fig. 1b.

Rat pup’s MR spectra were included based on spectral quality, using the following criteria as reported by LCModel (full width at half maximum ≤0.1 ppm, signal-to-noise ratio ≥6).^35^ Scans with obvious artifacts due to gross motion and poor water suppression were excluded. Using explicit formulas for LCModel, a single water attenuation coefficient was calculated and implemented when processing all spectra. We did not make partial volume corrections in these immature animals with small cerebrospinal fluid spaces and largely uniform brain matter. The following metabolites were analyzed for absolute concentrations in the right hemisphere using a validated basis set for LCModel^34,36^ that included GSH (Glutathione), GLX (Glutamate+Glutamine), Cr (Phosphocreatine+Creatine), NAA (NAA+*N*-acetylaspartylglutamate), and Cho (Choline+Glycerophosphocholine). Major peaks for these metabolites are noted on a representative spectrum (Fig. 1c). Inclusion of metabolite concentrations into analysis was based on Cramer–Rao <15% for all metabolites. The range of Cramer–Rao bounds for lactate were outside of inclusion criteria. LCModel fitting of the spectra, evaluation of spectral quality, and quantification of metabolites were performed by a researcher blinded to the treatment group.

### Statistical analysis

Statistical analysis was performed using SPSS^©^ Statistics, version 24 (IBM^©^, Armonk, NY). A two-way analysis of variance (ANOVA) with Tukey post hoc analysis was performed on normally distributed data (Experiment 1). In Experiment 2, non-parametric analyses, Wilcoxon signed-rank test and Kruskal–Wallis H Test, with pairwise post hoc analysis, and Spearman’s correlation were performed for behavioral scores and MRS metabolite concentrations, as data were not normally distributed and of unequal sample size. Significance was set at *p* < 0.05.

## Results

**Fig. 2:**
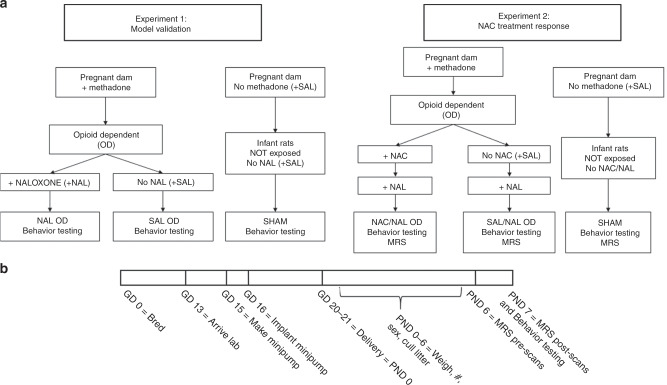
Experimental Design. **a** Flow diagram of within-litter randomization to treatment groups for Experiments 1 and 2. Experiment 1: Establish the antenatal methadone-dependent and neonatal acute withdrawal model. Treatment groups: NAL OD: methadone-exposed, opioid-dependent pups, naloxone-precipitated acute withdrawal; SAL OD: methadone-exposed, opioid-dependent pups, no naloxone; and Sham: saline exposed, no opioid dependence, no naloxone. Experiment 2: NAC effects on acute withdrawal behaviors and CNS metabolism. Treatment groups: NAC/NAL OD: methadone-exposed, opioid-dependent pups, NAC, naloxone-precipitated acute withdrawal; SAL/NAL OD: methadone-exposed, opioid-dependent pups, no NAC, naloxone-precipitated acute withdrawal; and Sham: saline exposed, no opioid dependence, no NAC, no naloxone. **b** Experiment timeline.

Randomized group assignments and timeline are shown in Fig. 2. Because of the time constraints for the MR scanner and the time needed to perform serial MRI scans, not all pups in a litter could undergo behavioral testing or be scanned at all time points.

Methadone exposure significantly blunts weight gain over the first week of life. Within Experiment 1, one-way ANOVA of the weight gain over 7 PNDs from birth to testing date (PND 7) for 3 groups was statistically significant (*F* = 12.5, *p* < 0.001). Tukey post hoc analysis showed that there were significant differences between Sham and both OD groups (both with *p* < 0.05): mean ± standard deviation NAL OD = 8.9 ± 1.7 g (*n* = 9), SAL OD 8.0 ± 2.0 g (*n* = 8), Sham 13.2 ± 0.9 g (*n* = 4). SAL OD and NAL OD were not different (*p* = 0.56), confirming that methadone exposure impedes normal postnatal weight gain. Similar effects of methadone on postnatal weight gain by group were seen in Experiment 2 (ANOVA: *F* = 13.1, *p* < 0.001). Tukey post hoc analysis confirmed that significant differences were only between Sham and OD groups (*p* < 0.05): NAC/NAL OD = 9.4 ± 0.7 g, *n* = 9; SAL/NAL OD = 8.6 ± 1.1 g, *n* = 10; Sham 11.3 ± 0.5 g, *n* = 4). OD SAL/NAL and OD NAC/NAL were not different (*p* = 0.39). Furthermore, mean ± standard deviation postnatal weight gain for the OD pups only was not statistically different between experiments (*p* = 0.1): combined OD groups in Experiment 1 = 8.4 ± 1.9 g (*n* = 17) and Experiment 2 = 9.0 ± 1.0 g (*n* = 19).

### Experiment 1: model validation

**Fig. 3:**
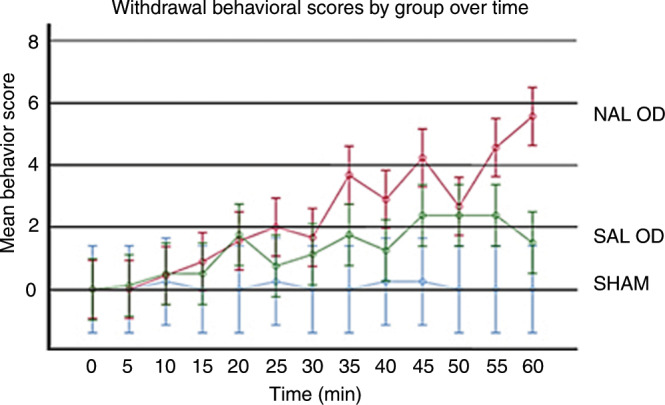
Mean ± SD withdrawal behavioral scores by group over time for Experiment 1. Analysis by two-way ANOVA for the model: *F* = 7.95, *p* < 0.001, by group: *F* = 47.05, *p* < 0.001, by time: *F* = 6.51, *p* < 0.001, and by group–time: *F* = 2.98, *p* < 0.001.

In the antenatal and postnatal model of methadone dependency and acute opioid withdrawal in the neonatal rat, methadone was delivered to the dam continuously by s.c. infiltration and to the pups for 11–12 days, as a combination of antenatal hematogenous and postnatal breast milk exposure. Naloxone elicited acute opioid withdrawal behaviors on PND 7: the mean ± standard deviation withdrawal behavioral scores for NAL OD = 30.1 ± 9.7 (*n* = 9), SAL OD = 16.4 ± 8.6 (*n* = 8), and Sham = 1.0 ± 1.4 (*n* = 4) (Fig. 3). Two-way ANOVA with repeated measures for independent variables of treatment group and time demonstrated strong significance for the model (*F* = 7.95, *p* < 0.001). Tukey post hoc analysis by group (NAL OD vs SAL OD; NAL OD vs Sham; SAL OD vs Sham) indicated that all comparisons were statistically significant (all *p* < 0.001). Behavioral scores for the SAL OD were significantly different from Sham scores, possibly due to mild withdrawal symptoms over time with less methadone in breast milk than exposure in utero.^13,27,28^ Further analysis demonstrated that a clear difference in behavioral scores was present as early as 35 min after NAL or SAL administration (*p* < 0.05).

### Experiment 2: NAC treatment response

#### NAC improves behavioral withdrawal scores

**Fig. 4:**
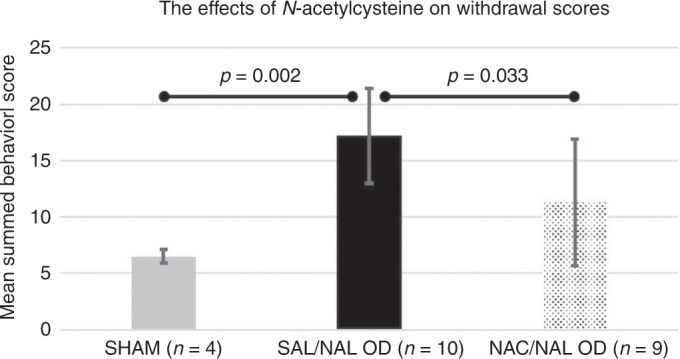
Comparison of mean summed behavioral scores (35–75 min) by group using Kruskal–Wallis H Test: *χ*^2^(2) = 10.49, *p* = 0.005, with pairwise post hoc analysis.

In Experiment 2, we observed the withdrawal behaviors for 90 min and then summed the behavioral scores from 35 to 75 min (the peak withdrawal time) after naloxone or saline. Similar to Experiment 1, there was a statistically significant difference in the mean summed behavioral scores for the model Kruskal–Wallis *H* Test: *χ*^2^ = 10.49, *p* = 0.005 (Fig. 4). Pairwise post hoc analysis showed that the SAL/NAL OD group mean withdrawal score (17.2 ± 4.2, *n* = 10) was significantly greater than the Sham group mean withdrawal score (6.5 ± 0.6, *n* = 4, *p* = 0.002). NAC significantly decreased the mean withdrawal score in the NAC/NAL OD group (11.3 ± 5.6, *n* = 9) compared to the SAL/NAL OD group (*p* = 0.033). Mean summed withdrawal scores in the NAC group were not significantly different from Sham (*p* = 0.14), indicating that NAC treatment mitigated the withdrawal behavioral seen in acute opioid withdrawal in the neonatal rat.

#### NAC mitigates oxidative stress of acute opioid withdrawal

**Fig. 5:**
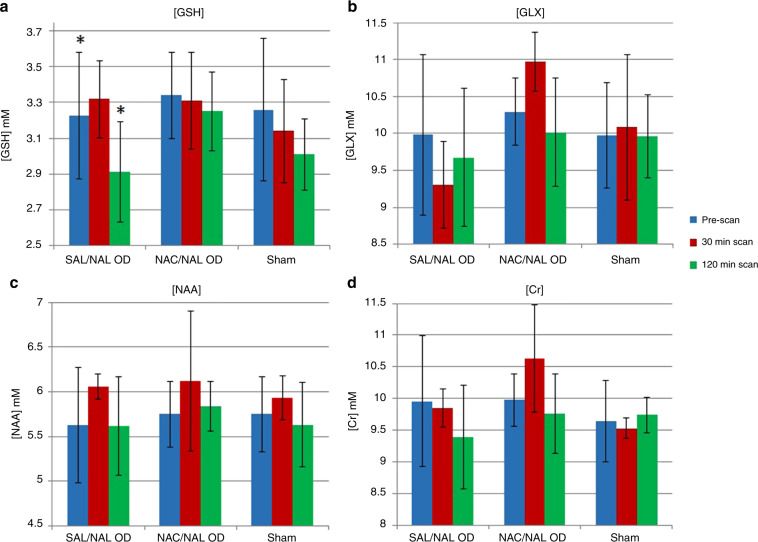
MRS metabolite concentrations (mM) in sham (Sham) and opioid-dependent rats receiving saline (SAL/NAL OD, n = 9) or NAC (NAC/NAL OD, n = 8) at pre- and post-withdrawal (30 and 120 min). **a** [GSH] Glutathione; **b** [GLX] Glutamate–Glutamine; **c** [NAA] *N*-acetylaspartate; **d** [Cr] Creatine. Comparison between pre-scan (blue bars) and 120-min scan (green bars) were done using Wilcoxon signed-rank test (*p < 0.05). There are no significant differences in the pre-withdrawal mean concentrations of any metabolite between groups.

To evaluate the effects of NAC on various CNS metabolites, we performed pre-withdrawal followed by serial (30 and 120 min) post-withdrawal MRS scans. Figure 5 shows the changes in GSH, GLX, NAA, and Cr over this period for the three groups (Sham, SAL/NAL OD, NAC/NAL OD). Owing to low sample size at the 30-min group (*n* = 3) and unequal distribution, within-group analysis was done using Wilcoxon signed-rank test comparing the pre-scans and 120-min post-withdrawal scans. In the SAL/NAL OD group (*n* = 9), [GSH] significantly decreased over the 120 min (*Z* = −2.55, *p* = 0.01) and administration of NAC in the NAC/NAL OD group (*n* = 8) at the time of acute precipitated withdrawal produced no significant difference in [GSH] over the 120-min (*Z* = −1.1, *p* = 0.26). When looking at [GLX], there was no statistically significant changes in either the SAL/NAL OD group (*Z* = −0.89, *p* = 0.37) or the NAC/NAL OD group (*Z* = −0.84, *p* = 0.40). Similarly, in regards to [NAA] and [Cr], there was no change in either metabolite over time with and without NAC ([NAA]: SAL/NAL OD group: *Z* = −0.3, *p* = 0.77; NAC/NAL OD group: *Z* = −0.70, *p* = 0.48; [Cr]: SAL/NAL OD group: *Z* = −1.2, *p* = 0.21; NAC/NAL OD group: *Z* = −0.7, *p* = 0.48). Sham rats showed no significant differences in any metabolites over time.

For between-group analysis, we performed Kruskal–Wallis test for pre-withdrawal (PND 6/7) and 120-min scans. For Sham, we combined 30- and 120-min metabolites to reach *n* = 8 observations, as they were not significantly different by time after saline. Before withdrawal on PND 6/7, there were no significant differences in GSH nor any other metabolites between groups. At 120 min, only GSH showed significant differences between groups (Kruskal–Wallis *H* statistic = 6.7, *p* = 0.035). At 120-min after naloxone, CNS GSH concentrations in the SAL/NAL OD group were significantly lower than in the NAC/ NAL OD group (*Z* = −2.38, *p* = 0.017, by Mann–Whitney), while [GSH] in the NAC/NAL OD rats were not significantly different than Sham. No other pairwise comparisons were significant at 120 min for GSH.

#### Withdrawal behavior correlate with CNS [GSH]

**Fig. 6:**
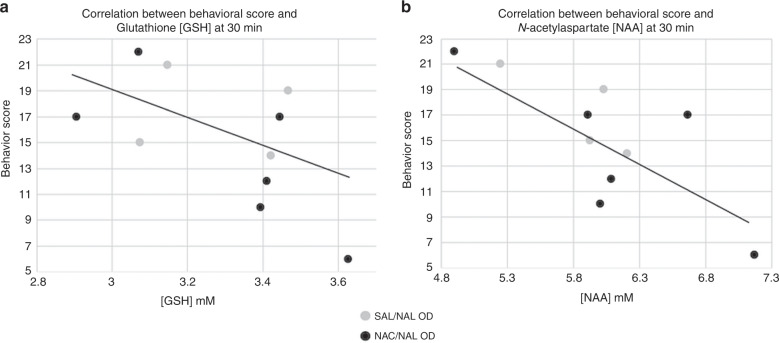
Withdrawal behavior scores and MRS metabolite concentrations at 30 min post-precipitated withdrawal. Gray circles represent the SAL/NAL OD group; black circles represent the NAC/NAL OD group. *n* = 10. **a** [GSH] vs behavioral scores (*r* = −0.60, *p* = 0.033); **b** [NAA] vs behavioral scores (*r* = −0.77, *p* = 0.005).

We assessed the relationship between mean summed behavioral scores and MRS metabolites. Mean summed behavioral scores of all withdrawal groups (NAC/NAL OD and SAL/NAL OD, *n* = 10) were plotted against the 30- and 120-min MRS metabolites. There was a significant negative correlation between behavior scores and 30-min [GSH] and [NAA] (Fig. 6). Lower CNS [GSH] and [NAA] 30 min after naloxone were associated with worse behavioral scores and greater symptoms of withdrawal. There was no statistically significant correlation seen between behavior score and 30-min [GLX] or [Cr] or any of the four CNS metabolites at 120 min post-withdrawal.

## Discussion

In these investigations, we established a model of antenatal opioid dependence and validated acute opioid withdrawal scoring in the neonatal rat in our laboratory. We demonstrated significantly different trajectories of behavioral withdrawal scores by the naloxone or saline groups over time and determined that withdrawal scores were still increasing at 60 min. We increased the withdrawal observation period to 90 min and captured the peak withdrawal period from 35 to 75 min. After co-administering NAC with naloxone, the NAC-treated opioid-dependent neonatal rats (NAC/NAL OD) had behavioral scores during acute withdrawal similar to the Sham pups (Fig. 4) and significantly lower behavioral scores than the SAL/NAL OD neonatal rats. Therefore, NAC mitigated behavioral effects of naloxone for at least 2 h after precipitating acute withdrawal. With equivalent weight gain between the OD groups, these effects were not due to dose effect from breast milk. NAC is a well-studied, Food and Drug Administration (FDA)-approved drug, with a favorable safety profile in neonates, giving these findings significant translational potential in the face of the opioid epidemic and rising rates of NAS after exposure to opioids in utero. Furthermore, NAC has been investigated as a safe neuroprotectant in animal models and human neonates with hypoxic ischemic injury, infection, and white matter injury.^22,34,37,38^ In addition, we have demonstrated that NAC can significantly increase [GSH] in the basal ganglia of neonates 5 days after hypoxic ischemic birth, mitigating ongoing oxidative stress.^34^

Cerebral oxidative stress has been well described during acute opioid withdrawal.^14–16^ Using in vivo MRS, we were able to demonstrate a significant decrease in [GSH] at 120 min after naloxone, compared with pre-withdrawal scans within the saline-treated group, and a significantly lower [GSH] at 120 min after naloxone in OD rats treated with saline vs NAC. Pre-withdrawal GSH concentrations were not significantly different between groups. As hypothesized, a single dose of NAC (50–100 mg/kg) completely inhibited the [GSH] decrease observed with naloxone and saline, and the protection persisted for at least 2 h. Therefore, at doses safely used even in sick neonates, NAC prevented CNS oxidative stress in acutely withdrawing, opioid-dependent neonatal rats.

NAC has been used extensively in addiction research to normalize glutamate homeostasis. However, we did not see an increase in glutamate–glutamine concentration pre-withdrawal between Sham and OD rat pups nor following precipitated withdrawal up to 120 min after one dose of NAC. This may be due to the measurement of the combined peak of glutamate and glutamine or the need for more time or a steady-state concentration of NAC to increase the activity of metabotropic glutamate receptors and to downregulate synaptic glutamate release.

Combining all opioid-dependent treatment groups, we found a significant association between CNS concentrations of [GSH] and [NAA] at 30 min and subsequent behavioral scores, summed over the following 40 min after the scan. The degree of early oxidative stress presaged the behavioral scores across all withdrawing neonatal rat groups. It is possible that longer behavioral observation would reveal that the [GSH] concentrations at the second post-treatment scan predict continuing withdrawal behavior beyond 120 min.

We also found that depletion of [NAA] correlated with worse behavioral scores. [NAA] is a marker of healthy neuronal metabolism and an energy substrate with donation of acetyl-CoA. [NAA] has been shown to rapidly decrease with cell injury and death after stroke^39^ and be one of the single best prognostic biomarkers after neonatal hypoxic ischemic encephalopathy. Our data suggest that [NAA] may also be a marker of CNS injury after opioid dependence and withdrawal.

Limitations of these studies include lack of sample size to analyze sex differences in metabolites, which have been described for CNS acetylcholine in an antenatal model of OD^40^ but not for behavioral scores after naloxone-precipitated withdrawal.^28^ Also, while there were no other significant changes over time or differences between treatment groups in total [Cr] or choline, our sample size may have been limited or the time periods not optimized for these metabolites. Subsequent doses of NAC may be required to demonstrate differences in chronic regulation of [GLX] and cellular energetics reflected in total [Cr].

## Conclusions

This is the first known study using NAC as a treatment for acute opioid withdrawal in the neonatal rat. As hypothesized, NAC mitigated behavioral withdrawal symptoms and oxidative stress in the opioid-dependent neonatal rat. NAC is FDA approved with known pharmacokinetics and pharmacodynamics in human neonates and could rapidly be translated into a Phase 1 clinical trial for the treatment of NAS.

## Supplementary information

Supplementary Table S1
