# Supplementary material for: N-acetylcysteine mitigates acute opioid withdrawal behaviors and CNS oxidative stress in neonatal rats
Source: Pediatr Res. 2020 Jan 14;88(1):77–84. doi: 10.1038/s41390-019-0728-6 (PMC7326708; doi:10.1038/s41390-019-0728-6)
Supplement: Supplementary file 1 — Supplementary Table S1 [file 41390_2019_728_MOESM1_ESM.docx]

Supplemental Table S1

| **Mean (SD)**  **Chamber Start Temperature ⁰C**  **n = 20** | **Mean (SD)**  **Chamber End Temperature ⁰C**  **n = 20** | **p-value*** |
| --- | --- | --- |
| 33.4 (0.9) | 33.6 (1.1) | 0.4 |
|  |  |  |
| **Mean (SD)**  **Body Start Temperature ⁰C**  **n = 29** | **Mean (SD)**  **Body End Temperature ⁰C**  **n = 29** | **p-value*** |
| 36.8 (0.3) | 36.9 (0.1) | 0.07 |
